# Dedicated CCTA Followed by High-Pitch Scanning versus TRO-CT for Contrast Media and Radiation Dose Reduction: A Retrospective Study

**DOI:** 10.3390/diagnostics12112647

**Published:** 2022-10-31

**Authors:** Kun Wang, Xiaodong Wang, Shaoqiang Zheng, Cheng Li, Liang Jin, Ming Li

**Affiliations:** 1 Radiology Department, Huadong Hospital Affiliated to Fudan University, Shanghai 200040, China; 2 Shanghai Changfeng Community Health Service Center of Putuo District, Shanghai 200062, China; 3 Department of Radiology, Tongji Hospital, School of Medicine, Tongji University, Shanghai 200065, China; 4 Institute of Functional and Molecular Medical Imaging, Fudan University, Shanghai 200040, China

**Keywords:** coronary CT angiography, high-pitch scanning, radiation dose, contrast media, image quality

## Abstract

We aimed to compare dedicated coronary computed tomography angiography (CCTA) followed by high-pitch scanning and triple-rule-out computed tomography angiography (TRO-CTA) in terms of radiation dose, contrast media (CM) use, and image quality. Patients with acute chest pain were retrospectively enrolled and assigned to group A (*n* = 55; scanned with dedicated CCTA followed by high-pitch scanning) or group B (*n* = 45; with TRO-CTA). Patient characteristics, radiation dose, CM use, and quantitative parameters (CT value, image noise, signal-to-noise ratio, contrast-to-noise ratio, and image quality score) of pulmonary arteries (PAs), thoracic aortae (TAs), and coronary arteries (CAs) were compared. The total effective dose was significantly lower in group A (6.25 ± 2.94 mSv) than B (8.93 ± 4.08 mSv; *p* < 0.001). CM volume was significantly lower in group A (75.7 ± 8.9 mL) than B (95.0 ± 0 mL; *p* < 0.001). PA and TA image quality were significantly better in group B, whereas that of CA was significantly better in group A. Qualitative image scores of PA and TA scans rated by radiologists were similar, whereas that of CA scans was significantly higher in group A than B (*p* < 0.001). Dedicated CCTA followed by high-pitch scanning demonstrated lower radiation doses and CM volume without debasing qualities of PA, TA, and CA scans than did TRO-CTA.

## 1. Introduction

Over 8 million people visit emergency departments owing to chest pain yearly [1,2]. Chest pain remains a major diagnostic challenge in emergency departments [3]. The primary concern of emergency departments is to eliminate potential life-threatening causes of chest pain, pulmonary embolism (PE), aortic dissection (AD), and acute coronary syndrome (ACS) [4]. Triple-rule-out computed tomography angiography (TRO-CTA) has been used because of its rapid results, non-invasiveness, and superiority. It also allows the simultaneous imaging of the pulmonary arteries (PAs), thoracic aortae (TAs), and coronary arteries (CAs) [3].

However, TRO-CTA is often associated with a higher radiation dose and increased use of contrast media (CM), although it achieves a slightly higher diagnostic yield for PE and AD [5,6]. For example, 120 kV tube voltage was used on dual-source CT with an effective dose of as high as 32.6 mSv [7]; in contrast, coronary computed tomography angiography (CCTA) showed evidence of DNA damage with an effective dose of over 7.5 mSv [8]. Additionally, a large volume of iodinated CM ranging from 80 to 120 mL in TRO-CTA is usually needed to maintain good image quality [6,9,10,11]. Although the rapid injection of high-dose iodinated CM enhances the visualization of the right atrium and right ventricle artifacts, it can seriously affect the observation of the right coronary artery.

Despite the above concerns, the optimal dose or optimal TRO-CTA protocol remains unclear [5]. Therefore, this study aimed to compare dedicated CCTA followed by high-pitch scanning and traditional TRO-CTA performed in patients with chest pain to evaluate the image quality, radiation dose, and use of iodinated CM.

## 2. Methods

### 2.1. Patient Population

A total of 104 patients with acute chest pain in our hospital between January 2021 and January 2022 were retrospectively enrolled. Patients aged ≥18 years with acute chest pain who were clinically suspected of having ACS, PE, or AD were included. The exclusion criteria were as follows: (1) lack of patient information (height, weight, heart rate) or (2) lack of scanning parameters, such as dose-length product (DLP), injection delivery rate, and volume of CM.

**Figure 1:**
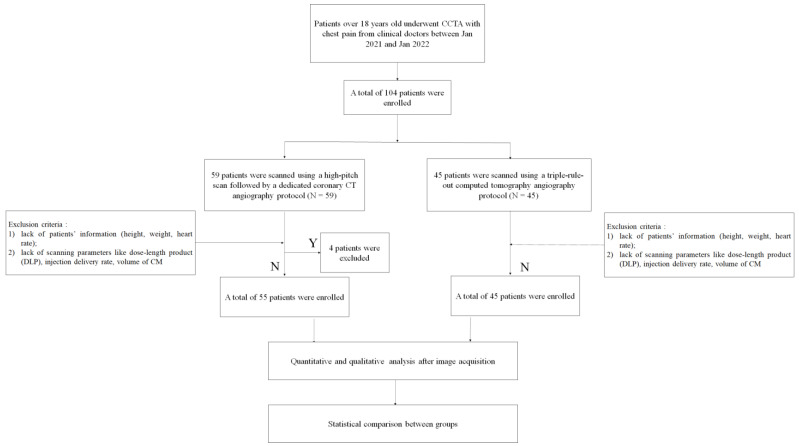
Flowchart of patient enrolment.

This study was approved by the ethics committee of our hospital, and the need for informed consent was waived. Finally, 100 patients were enrolled and divided between group A (*n* = 55) and group B (*n* = 45) (Figure 1).

### 2.2. Image Acquisition

All patients underwent imaging using a new generation of dual-source CT scanners (Somatom Drive, Siemens Healthcare, Forchheim, Germany). Dedicated CCTA followed by high-pitch scanning was used to scan the CAs first followed by the PAs and TA of the patients in group A. A calcium score (CS) was calculated to plan the scan range of CCTA using the following parameters: tube voltage, Sn100; automated anatomical tube current modulation, 268 mAs_ref.qual_ (CARE Dose 4D, Siemens Healthineers, Forchheim, Germany); and data acquisition window (R-R interval), 70%. For CCTA imaging, the collimation was 2 × 64 × 0.6 mm, and the rotation time was 0.28 s per rotation. A prospectively ECG-triggered sequence acquisition mode (step-and-shoot) was used, and scanning was performed from the cranium to the cauda. An automated anatomical tube current modulation technique with 380 mAs_ref.qual_ (CARE Dose 4D, Siemens Healthineers) and an automatic tube voltage selection with 120 kV_ref.qual_ (ATVS, CARE kV^TM^, Siemens Healthineers) were used, with the range of exposure dose (ECG-pulsing) set at 30–80%. A high-pitch scan or double high-pitch scan was performed to scan the entire chest in a cranial-to-caudal direction using an AVTS with 120 kV_ref.qual_ and a CARE Dose 4D with 100 mAs_ref.qual._, with a pitch of 2.2. The bolus tracking technique was used for threshold monitoring at the aortic root for CAs with an enhancement threshold of 80 HU and a delay time of 7 s, and for threshold monitoring at the left atrium with an enhancement threshold of 120 HU and a delay time of 4 s. Pre-warmed CM (iobitridol, 350 mg iodine [mgI]/mL) was injected using an 18-G closed intravenous catheter system with an Ulrich high-pressure syringe. For CCTA, the CM was administered according to the patient’s weight (0.8 mL/kg with a 13 s injection duration), and saline was administered at the same delivery rate with a 10 s injection duration. For PE and AD, the CM and saline were fixed at 25 mL and 40 mL, respectively, with a fixed delivery rate of 4 mL/s.

A retrospective ECG-triggered acquisition mode with a pitch of 0.17 was used to scan the entire chest in a cranial-to-caudal direction for patients in group B. An automated anatomical tube current modulation technique with 290 mA (CARE Dose 4D, Siemens Healthineers) and an automatic tube voltage selection with 100 kV_ref.qual_ (ATVS, CARE kV^TM^, Siemens Healthineers) was used. The exposure dose (ECG-pulsing) range was set at 35–80% for the R-R interval. The bolus tracking technique was used for threshold monitoring at the aortic root, with an enhancement threshold of 100 HU and a delay time of 9 s. Pre-warmed CM (iobitridol, 350 mg iodine [mgI]/mL) was injected using an 18-G closed intravenous catheter system with an Ulrich high-pressure syringe. The CM was administered in two phases: a fixed 50 mL of CM at a delivery rate of 4 mL/s and a fixed 30 mL of saline at a delivery rate of 3.5 mL/s were delivered immediately followed by the first phase using a fixed 45 mL of CM delivered at a delivery rate of 4.5 mL/s.

### 2.3. Image Reconstruction and Evaluation

In group A, the slice thickness and image reconstruction interval with the advanced modeled iterative reconstruction (strength level 4) algorithm for CAs were both 0.6 mm, and the kernel used was I26f. The slice thickness and image reconstruction interval with the advanced modeled iterative reconstruction (strength level 3) algorithm for the PE and aorta were both 1.0 mm, and the kernel used was I30f. In group B, the slice thickness and image reconstruction interval with the advanced modeled iterative reconstruction (strength level 3) algorithm for CAs were both 0.6 mm, and the kernel used was I26f. The slice thickness and image reconstruction interval with the advanced modeled iterative reconstruction (strength level 3) algorithm for PE and aorta was both 1.0 mm, and the kernel used was I26f.

The CT attenuation value and standard deviation (SD) of the pulmonary trunk (PT), left pulmonary artery (LPA), right pulmonary artery (RPA), aortic root (AO), aortic arch (AA), descending aorta (DA), proximal left main coronary artery (LMCA-P), middle left anterior descending (LAD-M), distal left anterior descending (LAD-D), middle left circumflex (LCX-M), distal left circumflex (LCX-D), proximal right coronary artery (RCA-P), middle right coronary artery (RCA-M), distal coronary right artery (RCA-D), erector spinae muscle (ESM), and perivascular adipose tissue (PVAT) were measured on the axial images using a region of interest of 20–400 mm^2^, and the contrast-to-noise ratio (CNR) for vessels was calculated. For the PAs and aorta, CNR was calculated using muscle as the background with the SD of muscle as the background noise; for CAs, fat was used as the background with the SD value of fat as the background noise.

The qualitative image score was assessed blindly and independently by two radiologists (one with eight years of experience in chest imaging, and the other with over 15 years of experience in chest imaging). The image quality score of CAs was based on the 15-segment classification by the modified American Heart Association classification [12]. PAs and TAs were scored using a four-point scoring system as follows: excellent (a score of 1) was defined as the complete absence of motion artifacts, excellent signal-to-noise ratio, and clear delineation of vessel walls, with the ability to assess luminal stenosis and plaque characteristics; good (score of 2) was defined as nonlimiting motion artifacts, reduced signal-to-noise ratio, and/or presence of calcifications, with the preserved ability to assess luminal stenosis and plaque characteristics; adequate (a score of 3) was defined as reduced image quality due to any combination of noise, motion, poor contrast enhancement, or presence of calcium that significantly impaired ease of interpretation but where the image quality was sufficient to rule out significant stenosis; and nondiagnostic (a score of 4) was defined as reduced image quality that precludes adequate assessment of stenosis in the majority of vessels [6].

### 2.4. Radiation Dose

The radiation doses of CS scan, CCTA, PAs, and aorta were recorded; the radiation associated with the scout view, CS scan, and the automatic bolus tracking technique was not included. The DLP and volume CT dose index (CTDIvol) were automatically provided by the CT scanner. The effective dose (ED) was estimated by multiplying the DLP by a conversion factor of 0.017 mSv/(mGy × cm) for the chest [13] and 0.026 for the CAs [8].

### 2.5. Statistical Analysis

The statistical software used was SPSS 22.0 (IBM, Chicago, IL, USA). Quantitative data are expressed as the mean ± SD (x ± s) or median (minimum, maximum), and count data are expressed as absolute values and percentages. If the general data of the groups, the objective evaluation indicators of image quality, and the radiation dose conformed to the normal distribution, the independent sample *t*-test was used. If the normal distribution was not confirmed, the non-parametric rank-sum test (Mann–Whitney U-test), the subjective scores of image quality, and diagnostic accuracy were compared using the chi-squared test. The consistency of the subjective scores of the two physicians was tested using the Kappa value. A Kappa value of 0.21–0.40 indicates poor consistency, 0.41–0.60 indicates moderate consistency, and 0.61–0.80 indicates good consistency. *p* < 0.05 indicates statistical significance.

## 3. Results

A total of 100 patients were included in this study, of whom 55 patients was enrolled in group A and 45 in group B (Figure 1). There were no significant differences in sex, age, height, weight, body mass index, and heart rate between the two groups (*p* > 0.05).

In group A, the tube voltage for CS was Sn100; for CCTA, 2, 3, 9, and 41 patients were scanned using 120 kV, 110 kV, 100 kV, and 90 kV, respectively. For the high-pitch scan, 12 patients were scanned using a double high-pitch scan for both PA and TA evaluation, whereas the other 43 patients were scanned using only a single high-pitch scan; the tube voltage is shown in Table 1. The CTDIvol and DLP for groups A and B are shown in Table 2. The total ED in group A was significantly lower (6.25 ± 2.94 mSv; *p* < 0.001) than that in group B (8.93 ± 4.08 mSv). The volume of CM in group A was significantly lower (75.7 ± 8.9 mL; *p* < 0.001) than that in group B (95.0 ± 0 mL), while the saline volume in group B was significantly lower (*p* < 0.001). The details are listed in Table 2 and Table 3.

The CT attenuation values of PT, LPA, RPA, AO, AA, DA, LMCA-P, LAD-D, LCX-D, RCA-P, and ESM in group A were significantly different from those in group B (*p* < 0.05). The image noise of PT, LPA, RPA, AO, AA, and DA in group A was significantly higher than that in group B (*p* < 0.05), whereas that of LMCA-P, LAD-M, LAD-D, LCX-M, LCX-D, RCA-P, RCA-M, and RCA-D in group A was significantly lower than that in group B (*p* < 0.05). The SNR values of PT, LPA, RPA, AO, AA, and DA in group B were significantly better than those in group A, whereas those of LAD-M, LAD-D, LCX-D, and RCA-M in group A were significantly better than those in group B (*p* < 0.05). The CNR values of PT, LPA, RPA, AO, AA, and DA in group B were significantly better than those in group A, whereas those of LMCA-P, LAD-D, and LCX-D in group A were significantly better than those in group B (*p* < 0.05).

**Figure 2:**
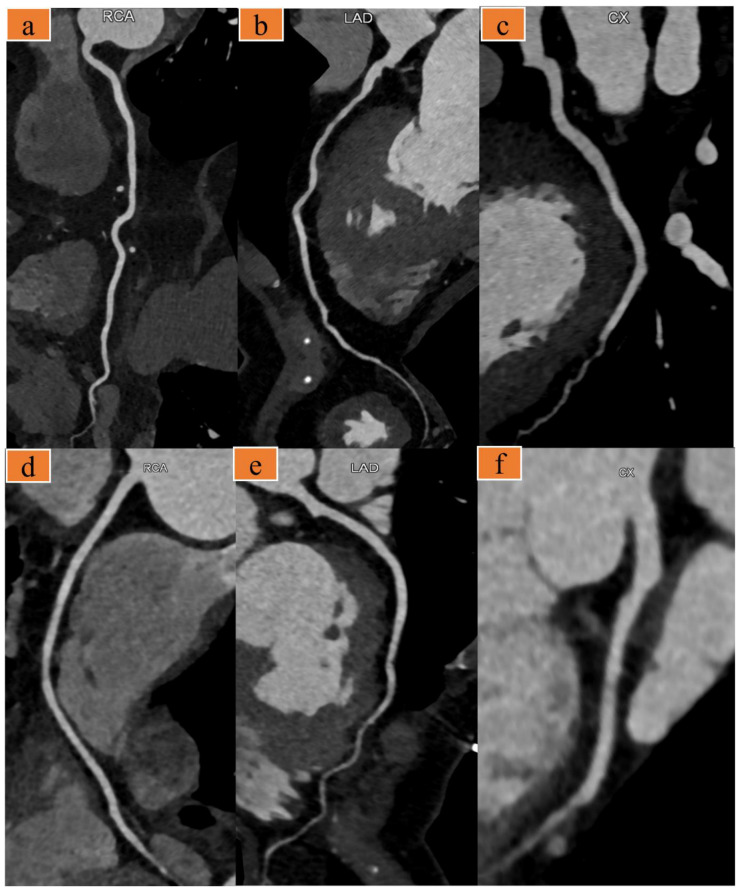
(**a**–**c**) A 91-year-old man with chief complaint of chest pain (heart rate: 67 bpm). Images were acquired with the optimized scanning scheme. Curved multiplanar reformat computed tomography (CT) images show the right coronary artery (**a**), left anterior descending artery (**b**), and left circumflex artery (**c**). (**d**–**f**) A 63-year-old woman presented to the emergency room with several days of chest pain, heart rate 58 bpm. Images were acquired with the traditional triple-rule-out computed tomography angiography (TRO-CTA). Curved multiplanar reformat CT images of the right coronary (**d**), left anterior descending artery (**e**), and left circumflex artery (**f**).

**Figure 3:**
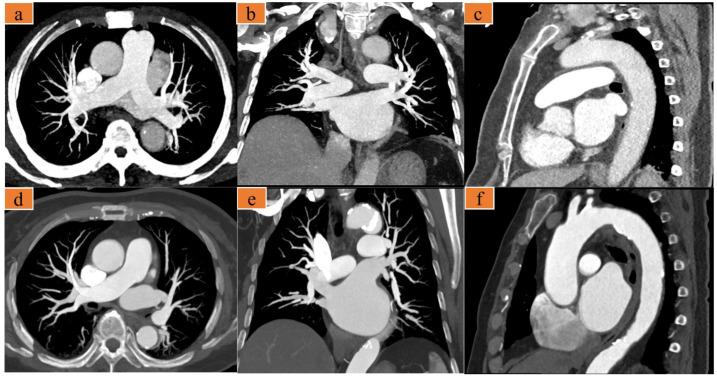
(**a**–**c**) A 75-year-old woman with chest pain, heart rate 77 bpm. Images were acquired with the optimized scanning scheme. (**d**–**f**) A 67-year-old woman presents to the emergency room with several days of chest pain, heart rate 66 bpm. Images were acquired with the traditional triple-rule-out computed tomography angiography (TRO-CTA). (**a**,**d**) are axial maximum intensity projection (MIP) images showing the pulmonary artery; (**b**,**e**) are coronal MIP images showing the pulmonary artery; and (**c**,**f**) are sagittal MIP images showing the thoracic aorta.

There was good inter-rater reliability between the two radiologists for the qualitative image score (κ = 0.89), and their average scores were used for further analysis. The qualitative image score showed that there was no significant difference in the PAs and CAs between groups, whereas the score for TAs in group A was significantly better than that in group B (Table 4, Figure 2 and Figure 3).

## 4. Discussion

Our study demonstrated the feasibility of a dedicated CCTA imaging followed by a high-pitch scan to achieve better image quality and lower radiation dose and CM volume than one-scan TRO-CTA. Our study also revealed an alternative for TRO-CTA using a dual-source CT. Compared with one-scan TRO-CTA, this technique significantly reduces the radiation dose and the CM volume without debasing the image quality.

Burris et al. reported that the use and appropriateness of TRO-CTA in the clinical setting must be further defined because the higher benefits of PE and AD were accompanied with a higher radiation dose and CM volume [6,13,14]. Only a few studies reporting the optimal protocol for both radiation dose and CM use using dual-source CT have been conducted after Burris et al.’s study. In 2017, Si-Mohamed et al. demonstrated that the second generation dual-source CT using a 100-kVp combing sinogram affirmed the iterative reconstruction algorithm for TRO-CTA reduced the ED to 5.7 ± 2.7 mSv using a conversion factor of 0.017 mSv/(mGy × cm) [9]. Compared with their study, our study achieved a slightly higher radiation dose (6.25 ± 2.94 mSv vs. 5.65 ± 1.37 mSv) but better image quality and lower CM volume. In 2018, He et al. demonstrated a surprisingly lower ED of 2.67 ± 0.98 mSv using a conversion factor of 0.014 mSv/(mGy × cm) but with a 16-cm wide-detector CT [13]. Compared with He et al.’s study with less exposure time of the 16-cm coverage detector [13,15], our study demonstrated that TRO-CTA could be achieved by a dedicated step-and-shoot CCTA that combines the short scan time of high-pitch scan; both of them were widely reported to achieve a lower radiation dose with good image quality [16,17,18,19], and the step-and-shoot CCTA with AVTS has been adaptive to higher heart rate [20,21]. In addition, the tube voltage at Sn100 of CS reportedly achieves a lower radiation dose without influencing the accuracy of the calcium score [22,23].

For CM use, Takx et al. [10] injected 120 mL of CM (Imeron 400 mgI/mL) using a dual-source CT system; Si-Mohamed et al. injected 90 mL (Imeron 400 mgI/mL) using a single source 64-section CT system; and another study injected 113 ± 16 mL, even including 57.4% scanned using a high-pitch scan [6]. Our study demonstrated the second lowest CM volume (75.0 ± 9.0 mL), which was slightly more than that (73.9 ± 7.4 mL) of He et al.’s study [13], whereas the 16-cm coverage detector prioritizes less exposure time with wide coverage compared with the 64-row CT system [13,15]. Although the volume of CM in contrast-enhanced CT does not harm the patients, catheter-based angiography after CTA with CM may increase the burden on the kidneys [24]. Our results showed that CT values, image noise, SNR, and CNR of the PAs and TAs in group B were significantly better than those in group A, but group A obtained lower radiation dose and lower CM volume from the high-pitch scans. Previous studies have shown that the CT values for PE and AD diagnosis were suggested to be more than 200 HU [25] and 250 HU [26], respectively, which may also explain the similar qualitative image score (*p* > 0.05, Table 4). CT values, image noise, SNR, and CNR of CA in group A were significantly better than those in group B, which could be proven with better qualitative image scores (2 vs. 3, Table 4), indicating that our method succeeded in obtaining a better image quality with CCTA imaging than PA and TA imaging. Compared with previous studies [9,13], the CNR and SNR of group A were also better. TRO-CTA was reported to be associated with a slightly higher incidence of PE and AD along with a much higher radiation and contrast dose [6], while there was not enough evidence to compare TRO-CTA with pulmonary CTA or aorta CTA for the diagnosis of PE or AD [5]. When TRO-CTA instead of CCTA was chosen in the emergency department, PE and AD were rarely found together [5,14]. Based on the above, our study tried to prioritize the quality of CCTA while appropriately debasing the image quality for PE and AD to meet the balance between image quality, radiation, and CM dose compared with that of the one-scan TRO-CTA. Our study demonstrated that better CCTA imaging combined with slightly worse PA and TA imaging with lower ED and CM use achieved similar image quality compared with one-scan TRO-CTA.

This study has some limitations. First, this single-center study had a small sample size, and the retrospective nature of the study design could not distinguish how the patients were enrolled into different groups. Moreover, the history of the patients was unknown; however, the patient characteristics between the groups showed no difference, which could support our results. Second, our study did not compare the diagnostic accuracies of PE, AD, and ACS because for all patients with negative results for PE, AD, and coronary angiography, the diagnostic accuracy of ACS could not be achieved without performing coronary angiography, although ACS was diagnosed in both groups. Further studies should take this into account. Finally, the radiation dose of CCTA in group A remained higher owing to the wide (35–80% for the R-R interval) exposure dose (ECG-pulsing) range.

In conclusion, dedicated CCTA followed by a high-pitch scan could achieve better image quality and similar image quality of PAs and TAs with lower radiation and contrast doses than the traditional one-scan TRO-CTA.

## Figures and Tables

**Table 1:** Tube voltage distribution.

	Group A				Group B
	CS	CCTA	High-Pitch1	High-Pitch2	
Sn100 kV	55	0	0	0	0
120 kV	0	2	3	1	1
110 kV	0	3	2	1	4
100 kV	0	9	4	0	9
90 kV	0	41	15	2	31
80 kV	0	0	31	8	0

CS, Calcium Score; CCTA: Coronary Computed Tomography Angiography. High pitch1: high pitch scan for pulmonary arteries. High pitch2: high pitch scan for aorta.

**Table 2:** Comparison of radiation dose parameters between two different TRO-CTA protocols.

Parameters	Group A	Group B	*p*
CTDIvol (mGy)	0.70 ± 0.38 (for CA) 18.75 ± 12.95 (for CCTA) 2.54 ± 1.56 (for high-pitch scan)	20.42 ± 9.60	0.516
DLP (mGy-cm)	8.81 ± 4.15 (for CA) 179.50 ± 94.48 (for CCTA) 79.86 ± 51.70 (for high-pitch scan)	525.28 ± 240.12	<0.001
ED (mSv)	0.23 ± 0.11 (for CA ∗ 0.026) 4.67 ± 2.46 (for CCTA ∗ 0.026) 1.36 ± 0.88 (for high-pitch scan ∗ 0.017)	8.93 ± 4.08 (∗0.017)	
Total ED (mSv)	6.25 ± 2.94	8.93 ± 4.08	<0.001
Delivery rate CM	3.90 ± 0.67 (for CCTA)	4.50 ± 0 (for first phase)	NA
	4.00 ± 0 (for high-pitch scan)	4.00 ± 0 (for second phase)	NA
Delivery rate Sa	3.90 ± 0.67 (for CCTA)	3.50 ± 0	NA
	4.00 ± 0 (for high-pitch scan)		
Contrast media (mL)	75.7 ± 8.9	95.0 ± 0	<0.001
Saline (mL)	79.0 ± 6.7	30.0 ± 0	<0.001

CTDIvol, CT dose index volume; DLP, dose-length product; ED, effective dose; TRO-CTA, triple-rule-out computed tomography angiography. ∗ for multiplication.

**Table 3:** Characteristics of patients examined by two different scan mode TRO-CTA protocols.

Criteria	Group A (*n* = 55)	Group B (*n* = 45)	*p*
Baseline characteristics			
Female (%)	30 (54.5%)	21 (46.7%)	0.433
Age (years)	66.6 ± 13.1 (23–91)	62.3 ± 10.6 (30–83)	0.076
Body mass index (kg/m^2^)	23.61 ± 3.18 (16.26–30.70)	24.35 ± 3.43 (17.29–31.85)	0.268
Heart rate (bpm)	73 ± 10 (51–92)	70 ± 10 (50–100)	0.173
Clinical outcomes			
Pulmonary embolism	0	0	
Aortic enlargement	0	3	
Pneumonia	5	3	
Aortic dissection	0	0	
Atherosclerotic ulcer	34	28	
Coronary artery stenosis	24	20	
Myocardial bridge	18	21	
Anomalous coronary artery	3	0	
PCI	3	2	

PCI: percutaneous coronary intervention, TRO-CTA: triple-rule-out computed tomography angiography.

**Table 4:** Results of the objective quality analysis.

Criteria	Group A (*n* = 55)	Group B (*n* = 45)	*p*
Attenuation values (HU)			
PT	346.95 (263.61, 476.55)	497.10 (391.98, 581.58)	<0.001
LPA	337.95 (256.95, 470.63)	470.84 (368.34, 535.67)	<0.001
RPA	357.14 (260.91, 471.70)	457.31 (359.58, 580.03)	<0.001
AO	321.67 (251.66, 385.13)	499.37 (418.23, 553.13)	<0.001
AA	300.62 (238.69, 375.24)	532.42 (456.91, 572.67)	<0.001
DA	262.38 (220.86, 359.63)	504.71 (440.26, 564.55)	<0.001
LMCA-P	441.50 (391.40, 501.11)	504.75 (443.31, 568.06)	0.001
LAD-M	376.92 (320.38, 434.67)	358.86 (288.77, 439.29)	0.201
LAD-D	305.88 (255.40, 363.30)	188.64 (147.35, 268.69)	<0.001
LCX-M	383.33 (329.55, 448.17)	399.47 (309.85, 485.99)	0.577
LCX-D	329.21 (255.27, 372.67)	260.50 (199.00, 305.05)	0.001
RCA-P	451.86 (395.00,510.20)	506.67 (417.81, 555.14)	0.040
RCA-M	469.92 (385.50,535.30)	471.45 (379.58, 529.00)	0.898
RCA-D	430.67 (350.15, 492.70)	454.19 (377.26, 526.09)	0.194
ESM	67.25 (57.33, 74.89)	50.83 (35.62, 57.98)	<0.001
PVAT	−107.30 (−116.45, −97.60)	−104.54 (−106.66, −100.60)	0.091
Image noise			
PT	26.46 (24.12, 30.42)	13.20 (11.49, 15.68)	<0.001
LPA	28.24 (24.50, 32.30)	14.18 (11.20, 16.34)	<0.001
RPA	27.12 (23.66, 31.90)	17.71 (14.59, 22.30)	<0.001
AO	30.08 (25.49, 33.58)	16.30 (12.81, 17.80)	<0.001
AA	22.36 (19.71, 24.66)	11.50 (10.33, 13.84)	<0.001
DA	28.74 (25.43, 31.16)	12.97 (11.31, 17.09)	<0.001
LMCA-P	10.59 (7.63, 13.51)	13.76 (9.65, 18.02)	0.007
LAD-M	17.28 (13.23, 21.13)	30.41 (19.41, 35.24)	<0.001
LAD-D	21.72 (16.27, 29.28)	30.42 (23.80, 42.19)	0.001
LCX-M	19.34 (12.35, 23.18)	21.58 (15.87, 33.36)	0.047
LCX-D	23.25 (15.87, 27.95)	30.28 (22.20, 37.79)	<0.001
RCA-P	12.44 (10.24, 14.04)	15.53 (11.79, 22.50)	0.001
RCA-M	15.49 (10.34, 20.49)	22.20 (15.10, 27.38)	0.002
RCA-D	20.27 (14.78, 26.23)	23.99 (18.29, 28.66)	0.043
SNR values			
PT	12.95 (10.57, 17.06)	37.95 (30.10, 44.15)	<0.001
LPA	13.07 (9.92, 15.26)	31.98 (26.61, 37.79)	<0.001
RPA	13. 80 (9.76, 15.91)	25.12 (20.66, 35.77)	<0.001
AO	10.67 (9.50, 12.96)	30.32 (25.28, 35.28)	<0.001
AA	13.28 (11.73, 17.26)	42.75 (35.39, 51.21)	<0.001
DA	9.64 (7.76, 13.14)	34.09 (27.31, 42.93)	<0.001
LMCA-P	39.83 (34.78, 54.37)	36.46 (27.44, 47.05)	0.128
LAD-M	21.87 (16.14, 29.90)	12.71 (8.86, 19.83)	<0.001
LAD-D	13.50 (10.69, 18.33)	6.18 (4.54, 9.27)	<0.001
LCX-M	20.70 (15.35, 29.37)	18.14 (9.41, 28.39)	0.167
LCX-D	14.14 (9.94, 21.53)	8.59 (5.77, 12.39)	<0.001
RCA-P	34.29 (25.59, 41.68)	28.83 (19.02, 44.13)	0.388
RCA-M	28.76 (21.23, 43.95)	21.97 (14.92, 32.56)	0.005
RCA-D	21.91 (15.17, 29.80)	18.76 (13.42, 26.92)	0.297
CNR values			
PT	41.47 (28.68, 60.64)	56.12 (44.90, 91.06)	0.001
LPA	40.93 (27.34, 59.47)	52.39 (39.65, 80.95)	0.004
RPA	40.85 (27.13, 60.76)	54.58 (39.57, 92.67)	0.004
AO	40.06 (26.77, 52.51)	56.47 (44.33, 90.85)	<0.001
AA	34.43 (24.68, 52.15)	60.57 (46.94, 95.05)	<0.001
DA	31.24 (21.58, 47.73)	59.94 (44.57, 93.00)	<0.001
LMCA-P	91.47 (74.58, 102.94)	99.20 (83.16, 114.09)	0.041
LAD-M	76.65 (67.14, 95.76)	73.75 (58.72, 89.71)	0.257
LAD-D	61.33 (54.40, 80.98)	48.23 (39.22, 59.04)	<0.001
LCX-M	79.11 (64.75, 96.09)	78.97 (67.42, 96.11)	0.790
LCX-D	70.57 (53.01, 84.02)	60.34 (46.78, 70.85)	0.020
RCA-P	89.32 (76.14, 106.14)	100.79 (82.20, 110.16)	0.187
RCA-M	88.51 (76.75, 107.25)	92.33 (73.68, 109.60)	0.893
RCA-D	83.21 (73.87, 99.20)	90.28 (71.59, 112.59)	0.456
Qualitative image score (4-point scale)			
Pulmonary arteries	2 (2, 2)	2 (1, 3)	0.199
Coronary arteries	2 (2, 2)	2 (2, 2.5)	0.101
Thoracic aorta	2 (2, 3)	2 (1, 2)	0.003

PT, pulmonary trunk; LPA, left pulmonary artery; RPA, right pulmonary artery; AO, Aortic root; AA, aortic arch; DA, descending aorta; SNR, signal-to-noise ratio; CNR, contrast-to-noise ratio; LMCA-P, proximal left main coronary artery; LAD-M, middle left anterior descending; LAD-D, distal left anterior descending; LCX-M, middle left circumflex; LCX-D, distal left circumflex; RCA-P, proximal right coronary artery; RCA-M, middle right coronary artery; RCA-D, distal coronary right artery; ESM, erector spinae muscle; PVAT, perivascular adipose tissue.

## Data Availability

The data underlying this article will be shared on reasonable request to the corresponding author.

## Abbreviations

**Abbreviation**	**Meaning**
TRO-CTA	Triple-Rule-Out Computed Tomography Angiography
CM	Contrast Media
CCTA	Coronary Computed Tomography Angiography
PA	Pulmonary Artery
TA	Thoracic Aorta
CA	Coronary Artery
PE	Pulmonary Embolism
AD	Aortic Dissection
ACS	Acute Coronary Syndrome
DLP	Dose-Length Product
CS	Calcium Score
ATVS	Automatic tube voltage selection
SD	Standard Deviation
PT	Pulmonary Trunk
LPA	Left Pulmonary Artery
RPA	Right Pulmonary Artery
AO	Aortic Root
AA	Aortic Arch
DA	Descending Aorta
LMCA-P	Proximal Left Main Coronary Artery
LAD-M	Middle Left Anterior Descending
LAD-D	Distal Left Anterior Descending
LCX-M	Middle Left Circumflex
LCX-D	Distal Left Circumflex
RCA-P	Proximal Right Coronary Artery
RCA-M	Middle Right Coronary Artery
RCA-D	Distal Coronary Right Artery
ESM	Erector Spinae Muscle
PVAT	Perivascular Adipose Tissue
SNR	Signal-to-Noise Ratio
CNR	Contrast-to-Noise Ratio
CTDIvol	CT dose index
ED	Effective Dose
